# *N* ^1^,*N* ^4^,3,6-Tetra­methyl-1,2,4,5-tetra­zine-1,4-dicarboxamide

**DOI:** 10.1107/S1600536812022519

**Published:** 2012-05-23

**Authors:** Na-Bo Sun, Guo-Wu Rao, Qun Shen

**Affiliations:** a College of Biology and Environmental Engineering, Zhejiang Shuren University, Hangzhou 310015, People’s Republic of China; b College of Pharmaceutical Science, Zhejiang University of Technology, Hangzhou 310014, People’s Republic of China; c Hangzhou Institute of Calibration and Testing for Quality and Technical Supervision, Hangzhou 310019, People’s Republic of China

## Abstract

The asymmetric unit of the title compound, C_8_H_14_N_6_O_2_, contains two independent mol­ecules. In one mol­ecule, the amide-substituted N atoms of the tetra­zine ring deviate from the plane [maximum deviation = 0.028 (1) Å] through the four other atoms in the ring by 0.350 (2) and 0.344 (2) Å, forming a boat conformation, and the mean planes of the two carboxamide groups form dihedral angles of 10.46 (13) and 20.41 (12)° with the four approximtely planar atoms in the tetra­zine ring. In the other mol­ecule, the amide-substituted N atoms of the tetra­zine ring deviate from the plane [maximum deviation = 0.033 (1) Å] through the four other atoms in the ring by 0.324 (2) and 0.307 (2) Å, forming a boat conformation, and the mean planes of the two carboxamide groups form dihedral angles of 14.66 (11) and 17.08 (10)° with the four approximately planar atoms of the tetra­zine ring. In the crystal, N—H⋯O hydrogen bonds connect mol­ecules to form a two-dimensional network parallel to (1-1-1). Intra­molecular N—H⋯N hydrogen bonds are observed.

## Related literature

For chemical reactions of 1,2,4,5-tetra­zine derivatives, see: Domingo *et al.* (2009 ▶); Lorincz *et al.* (2010 ▶) and for their biological activity, see: Devaraj *et al.* (2009 ▶); Eremeev *et al.* (1978 ▶, 1980 ▶); Han *et al.* (2010 ▶); Neunhoeffer (1984 ▶); Sauer (1996 ▶). For the anti­tumor activity of 1,2,4,5-tetra­zine derivatives, see: Hu *et al.* (2002 ▶, 2004 ▶); Rao & Hu (2005 ▶, 2006 ▶). For standard bond lengths, see: Allen *et al.* (1987 ▶). For the synthesis of the title compound, see: Hu *et al.* (2004 ▶); Rao *et al.* (2012 ▶); Sun *et al.* (2003 ▶).
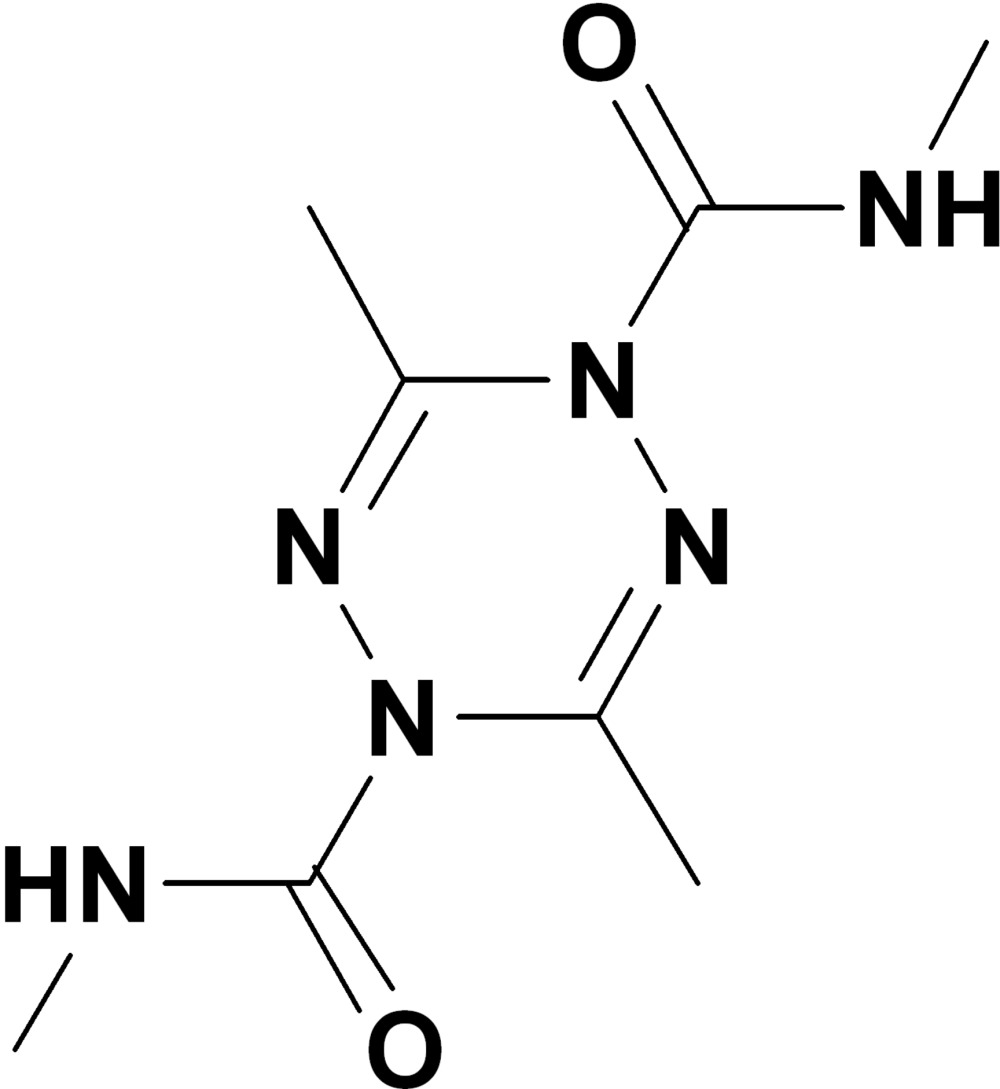


## Experimental

### 

#### Crystal data

C_8_H_14_N_6_O_2_
*M* *_r_* = 226.25
Triclinic, 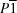

*a* = 9.0002 (17) Å
*b* = 12.045 (2) Å
*c* = 12.357 (2) Å
α = 118.386 (2)°
β = 101.701 (3)°
γ = 99.494 (3)°
*V* = 1099.6 (4) Å^3^
*Z* = 4
Mo *K*α radiation
μ = 0.10 mm^−1^
*T* = 298 K
0.34 × 0.30 × 0.15 mm

#### Data collection

Bruker SMART CCD diffractometer
Absorption correction: multi-scan (*SADABS*; Bruker, 1997 ▶) *T*_min_ = 0.966, *T*_max_ = 0.985
5571 measured reflections
3809 independent reflections
3178 reflections with *I* > 2σ(*I*)
*R*_int_ = 0.018

#### Refinement

*R*[*F* ^2^ > 2σ(*F* ^2^)] = 0.044
*wR*(*F* ^2^) = 0.115
*S* = 1.06
3809 reflections
298 parameters
H-atom parameters constrained
Δρ_max_ = 0.17 e Å^−3^
Δρ_min_ = −0.19 e Å^−3^

### 

Data collection: *SMART* (Bruker, 1997 ▶); cell refinement: *SAINT* (Bruker, 1997 ▶); data reduction: *SAINT*; program(s) used to solve structure: *SHELXS97* (Sheldrick, 2008 ▶); program(s) used to refine structure: *SHELXL97* (Sheldrick, 2008 ▶); molecular graphics: *ORTEP-3 for Windows* (Farrugia, 1997 ▶) and *PLATON* (Spek, 2009 ▶); software used to prepare material for publication: *WinGX* (Farrugia, 1999 ▶).

## Supplementary Material

Crystal structure: contains datablock(s) I, global. DOI: 10.1107/S1600536812022519/lh5475sup1.cif

Supplementary material file. DOI: 10.1107/S1600536812022519/lh5475Isup2.cdx

Structure factors: contains datablock(s) I. DOI: 10.1107/S1600536812022519/lh5475Isup3.hkl

Supplementary material file. DOI: 10.1107/S1600536812022519/lh5475Isup4.cml

Additional supplementary materials:  crystallographic information; 3D view; checkCIF report

## Figures and Tables

**Table 1:** Hydrogen-bond geometry (Å, °)

*D*—H⋯*A*	*D*—H	H⋯*A*	*D*⋯*A*	*D*—H⋯*A*
N3—H3⋯N2	0.86	2.16	2.572 (2)	108
N6—H6⋯N5	0.86	2.19	2.586 (2)	108
N9—H9⋯N8	0.86	2.15	2.567 (2)	109
N12—H12⋯N11	0.86	2.17	2.581 (2)	109
N3—H3⋯O4	0.86	2.20	2.925 (2)	142
N6—H6⋯O3^i^	0.86	2.16	2.918 (2)	146
N9—H9⋯O1^ii^	0.86	2.14	2.877 (3)	143
N12—H12⋯O2^iii^	0.86	2.24	2.967 (3)	142

Symmetry codes: (i) 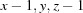; (ii) 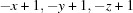; (iii) 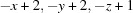.

**Fig. 1.**
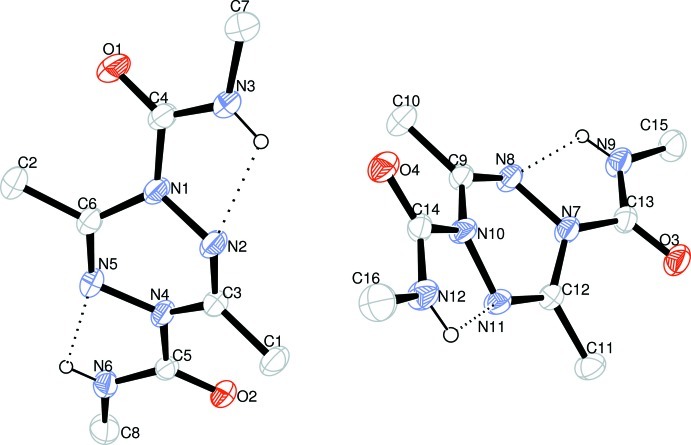
The molecular structure of (I), shown with 30% probability displacement ellipsoids. Hydrogen bonds are shown as dashed lines.

**Fig. 2.**
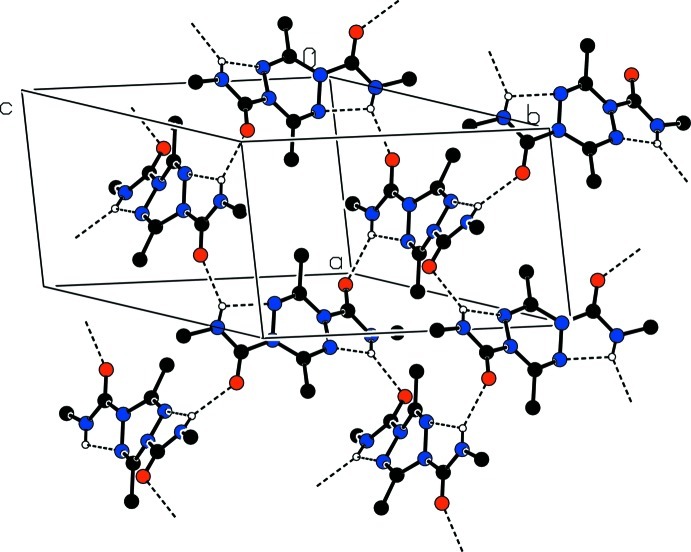
Part of the crystal structure with hydrogen donds shown as dashed lines.

## supplementary crystallographic information

### Comment

Tetrazine derivatives have high activity in chemical reactions (Domingo *et al.*, 2009; Lorincz *et al.*, 2010), and have been widely used in pesticides and medicines (Devaraj *et al.*, 2009; Eremeev *et al.*,1978, 1980; Han *et al.*, 2010; Neunhoeffer, 1984; Sauer, 1996). In a continuation of our studies of antitumor activities in 1,2,4,5-tetrazine derivatives (Hu *et al.*, 2002, 2004; Rao & Hu, 2005, 2006), we have obtained a colourless crystalline compound, (I). The structure was confirmed by single-crystal X-ray diffraction.

The two molecules forming the asymmetric unit of (I) are shown in Fig. 1. The C=N, N—N and C—N bonds have normal distances (Allen *et al.*, 1987). The tetrazine rings are 1,4-dihydro structure with the N-substituted groups at the 1,4-positions.

In (I), atoms N2, C3, N5 and C6 are approximately planar, with the largest deviation from this plane being 0.028 (1) Å. Atoms N1 and N4 deviate from this plane by 0.350 (2) and 0.344 (2) Å, respectively. Atoms N8, C9, N11 and C12 are approximately planar, with the largest deviation from this plane being 0.033 (1) Å. Atoms N7 and N10 deviate from this plane by 0.324 (2) and 0.307 (2) Å, respectively. The dihedral angle between the N2/C3/N5/C6 plane and the N1/N2/C6 plane is 27.99 (16)°, and between the N2/C3/N5/C6 plane and the N4/N5/C3 plane is 27.91 (16)°. The dihedral angle between the N8/C9/N11/C12 plane and the N7/N8/C12 plane is 26.36 (14)°, and between the N8/C9/N11/C12 plane and the N10/N11/C9 plane is 24.96 (13)°. The tetrazine ring exhibits a boat conformation. Atoms O1, C4, N3 and C7 are approximately planar, with the largest deviation from this plane being 0.016 (1) Å. Atoms O2, C5, N6 and C8 are approximately planar, with the largest deviation from this plane being -0.022 (1) Å. Atoms O3, C13, N9 and C15 are approximately planar, with the largest deviation from this plane being 0.004 (1) Å. Atoms O4, C14, N12 and C16 are approximately planar, with the largest deviation from this plane being -0.005 (1) Å. The dihedral angles between the N2/C3/N5/C6 plane and two planes of carboxamide groups are 10.46 (13) and 20.41 (12)°, respectively. The dihedral angles between the N8/C9/N11/C12 plane and two planes of carboxamide groups are 14.66 (11) and 17.08 (10)°, respectively. Intramolecular N—H···N hydrogen bonds are observed. In the crystal, N—H···O hydrogen bonds connect molecules to form a two-dimensional network parallel to (111) (Fig. 2).

### Experimental

The title compound was the product of the reaction of 3,6-dimethyl-1,6-dihydro-1,2,4,5-tetrazine, bis(trichloromethyl) carbonate and methanamine according to the procedure (Hu *et al.*, 2004; Rao *et al.*, 2012; Sun *et al.*, 2003). A solution of the compound in ethanol was concentrated gradually at room temperature to afford colourless blocks.

### Refinement

H atoms were included in calculated positions and refined using a riding model. H atoms were given isotropic displacement parameters equal to 1.2 (or 1.5 for methyl H atoms) times the equivalent isotropic displacement parameters of their parent atoms, and C—H distances were set to 0.96 Å for methyl H atoms, while N—H distances were set to 0.86 Å.

### Crystal data

**Table:**

C_8_H_14_N_6_O_2_	*Z* = 4
*M**_r_* = 226.25	*F*(000) = 480
Triclinic, *P*1	*D*_x_ = 1.367 Mg m^−^^3^
Hall symbol: -P 1	Mo *K*α radiation, λ = 0.71073 Å
*a* = 9.0002 (17) Å	Cell parameters from 2161 reflections
*b* = 12.045 (2) Å	θ = 2.4–26.0°
*c* = 12.357 (2) Å	µ = 0.10 mm^−^^1^
α = 118.386 (2)°	*T* = 298 K
β = 101.701 (3)°	Block, colourless
γ = 99.494 (3)°	0.34 × 0.30 × 0.15 mm
*V* = 1099.6 (4) Å^3^	

### Data collection

**Table:**

Bruker SMART CCD diffractometer	3809 independent reflections
Radiation source: fine-focus sealed tube	3178 reflections with *I* > 2σ(*I*)
Graphite monochromator	*R*_int_ = 0.018
φ and ω scans	θ_max_ = 25.0°, θ_min_ = 1.9°
Absorption correction: multi-scan (*SADABS*; Bruker, 1997)	*h* = −10→10
*T*_min_ = 0.966, *T*_max_ = 0.985	*k* = −14→11
5571 measured reflections	*l* = −11→14

### Refinement

**Table:**

Refinement on *F*^2^	Secondary atom site location: difference Fourier map
Least-squares matrix: full	Hydrogen site location: inferred from neighbouring sites
*R*[*F*^2^ > 2σ(*F*^2^)] = 0.044	H-atom parameters constrained
*wR*(*F*^2^) = 0.115	*w* = 1/[σ^2^(*F*_o_^2^) + (0.0511*P*)^2^ + 0.2386*P*] where *P* = (*F*_o_^2^ + 2*F*_c_^2^)/3
*S* = 1.06	(Δ/σ)_max_ < 0.001
3809 reflections	Δρ_max_ = 0.17 e Å^−^^3^
298 parameters	Δρ_min_ = −0.19 e Å^−^^3^
0 restraints	Extinction correction: *SHELXL97* (Sheldrick, 2008), Fc^*^=kFc[1+0.001xFc^2^λ^3^/sin(2θ)]^-1/4^
Primary atom site location: structure-invariant direct methods	Extinction coefficient: 0.028 (2)

### Special details

**Table:**

Geometry. All e.s.d.'s (except the e.s.d. in the dihedral angle between two l.s. planes) are estimated using the full covariance matrix. The cell e.s.d.'s are taken into account individually in the estimation of e.s.d.'s in distances, angles and torsion angles; correlations between e.s.d.'s in cell parameters are only used when they are defined by crystal symmetry. An approximate (isotropic) treatment of cell e.s.d.'s is used for estimating e.s.d.'s involving l.s. planes.
Refinement. Refinement of *F*^2^ against ALL reflections. The weighted *R*-factor *wR* and goodness of fit *S* are based on *F*^2^, conventional *R*-factors *R* are based on *F*, with *F* set to zero for negative *F*^2^. The threshold expression of *F*^2^ > σ(*F*^2^) is used only for calculating *R*-factors(gt) *etc*. and is not relevant to the choice of reflections for refinement. *R*-factors based on *F*^2^ are statistically about twice as large as those based on *F*, and *R*- factors based on ALL data will be even larger.

### Fractional atomic coordinates and isotropic or equivalent isotropic displacement parameters (Å^2^)

**Table:**

	*x*	*y*	*z*	*U*_iso_*/*U*_eq_	
O2	0.65578 (15)	1.05745 (12)	0.47718 (12)	0.0481 (3)	
O4	0.89425 (15)	0.52517 (12)	0.38657 (12)	0.0485 (3)	
O3	1.24324 (16)	0.92428 (15)	1.04950 (13)	0.0591 (4)	
O1	0.31715 (16)	0.38004 (14)	0.09001 (15)	0.0624 (4)	
N11	1.11229 (17)	0.83375 (14)	0.67258 (14)	0.0413 (4)	
N5	0.42071 (17)	0.76464 (15)	0.18158 (14)	0.0428 (4)	
N8	0.90988 (17)	0.73177 (15)	0.76534 (14)	0.0441 (4)	
N1	0.48121 (17)	0.59229 (14)	0.19776 (14)	0.0427 (4)	
N4	0.54445 (17)	0.84531 (14)	0.30278 (14)	0.0408 (4)	
N2	0.64314 (17)	0.67031 (15)	0.26959 (15)	0.0456 (4)	
N10	0.99591 (17)	0.70658 (13)	0.59312 (13)	0.0401 (4)	
N7	1.06880 (17)	0.81490 (15)	0.84255 (14)	0.0424 (4)	
C5	0.5569 (2)	0.98150 (17)	0.36969 (17)	0.0389 (4)	
C9	0.8816 (2)	0.67574 (17)	0.64282 (17)	0.0389 (4)	
N12	1.10612 (19)	0.69002 (15)	0.43553 (14)	0.0459 (4)	
H12	1.1731	0.7660	0.4950	0.055*	
C12	1.1485 (2)	0.88147 (16)	0.79504 (16)	0.0378 (4)	
N3	0.57057 (19)	0.42708 (15)	0.20694 (15)	0.0479 (4)	
H3	0.6588	0.4886	0.2591	0.058*	
C4	0.4495 (2)	0.45756 (18)	0.15889 (17)	0.0432 (4)	
N6	0.45245 (19)	1.01525 (15)	0.30820 (15)	0.0470 (4)	
H6	0.3919	0.9577	0.2297	0.056*	
N9	0.99787 (19)	0.79818 (17)	1.00444 (15)	0.0511 (4)	
H9	0.9073	0.7470	0.9458	0.061*	
C14	0.9928 (2)	0.63353 (17)	0.46360 (16)	0.0379 (4)	
C3	0.6682 (2)	0.79506 (18)	0.32349 (17)	0.0408 (4)	
C13	1.1104 (2)	0.85187 (18)	0.97372 (17)	0.0425 (4)	
C6	0.3891 (2)	0.64048 (18)	0.13459 (17)	0.0394 (4)	
C10	0.7187 (2)	0.5826 (2)	0.55809 (19)	0.0531 (5)	
H10A	0.6737	0.6075	0.4987	0.080*	
H10B	0.6527	0.5861	0.6110	0.080*	
H10C	0.7249	0.4941	0.5100	0.080*	
C11	1.2743 (2)	1.01175 (18)	0.88275 (18)	0.0505 (5)	
H11A	1.2369	1.0699	0.9493	0.076*	
H11B	1.2985	1.0501	0.8334	0.076*	
H11C	1.3685	0.9993	0.9221	0.076*	
C1	0.8344 (2)	0.88257 (19)	0.4028 (2)	0.0555 (5)	
H1A	0.8627	0.9432	0.3753	0.083*	
H1B	0.9055	0.8296	0.3914	0.083*	
H1C	0.8422	0.9314	0.4929	0.083*	
C2	0.2535 (2)	0.5519 (2)	0.01109 (18)	0.0531 (5)	
H2A	0.2876	0.4846	−0.0507	0.080*	
H2B	0.2182	0.6029	−0.0235	0.080*	
H2C	0.1676	0.5110	0.0279	0.080*	
C15	1.0255 (3)	0.8246 (2)	1.13515 (19)	0.0585 (6)	
H15A	0.9317	0.7778	1.1395	0.088*	
H15B	1.0496	0.9179	1.1949	0.088*	
H15C	1.1133	0.7955	1.1578	0.088*	
C7	0.5575 (3)	0.29262 (19)	0.1735 (2)	0.0622 (6)	
H7A	0.6410	0.2931	0.2361	0.093*	
H7B	0.5669	0.2428	0.0886	0.093*	
H7C	0.4561	0.2527	0.1736	0.093*	
C8	0.4394 (3)	1.1471 (2)	0.3715 (2)	0.0615 (6)	
H8A	0.3507	1.1515	0.3172	0.092*	
H8B	0.5354	1.2086	0.3867	0.092*	
H8C	0.4235	1.1694	0.4530	0.092*	
C16	1.1183 (3)	0.6249 (2)	0.30563 (19)	0.0606 (6)	
H16A	1.2061	0.6797	0.3028	0.091*	
H16B	1.0215	0.6108	0.2444	0.091*	
H16C	1.1354	0.5413	0.2838	0.091*	

### Atomic displacement parameters (Å^2^)

**Table:**

	*U*^11^	*U*^22^	*U*^33^	*U*^12^	*U*^13^	*U*^23^
O2	0.0450 (7)	0.0421 (7)	0.0381 (7)	0.0013 (6)	0.0023 (6)	0.0150 (6)
O4	0.0481 (8)	0.0373 (7)	0.0384 (7)	0.0026 (6)	0.0046 (6)	0.0109 (6)
O3	0.0435 (8)	0.0760 (10)	0.0429 (8)	−0.0041 (7)	−0.0020 (6)	0.0335 (8)
O1	0.0390 (8)	0.0510 (8)	0.0730 (10)	−0.0083 (7)	−0.0028 (7)	0.0297 (8)
N11	0.0412 (9)	0.0335 (8)	0.0358 (8)	0.0004 (6)	0.0062 (7)	0.0142 (7)
N5	0.0354 (8)	0.0437 (9)	0.0340 (8)	0.0057 (7)	0.0019 (7)	0.0148 (7)
N8	0.0333 (8)	0.0458 (9)	0.0382 (9)	0.0007 (7)	0.0040 (7)	0.0176 (7)
N1	0.0308 (8)	0.0387 (8)	0.0433 (9)	0.0011 (6)	0.0026 (7)	0.0170 (7)
N4	0.0337 (8)	0.0375 (8)	0.0354 (8)	0.0039 (6)	0.0007 (6)	0.0139 (7)
N2	0.0311 (8)	0.0403 (9)	0.0478 (9)	0.0012 (7)	−0.0003 (7)	0.0183 (7)
N10	0.0405 (8)	0.0330 (8)	0.0312 (8)	−0.0004 (6)	0.0054 (6)	0.0114 (6)
N7	0.0351 (8)	0.0463 (9)	0.0337 (8)	0.0013 (7)	0.0041 (6)	0.0184 (7)
C5	0.0347 (9)	0.0410 (10)	0.0344 (10)	0.0026 (8)	0.0086 (8)	0.0189 (8)
C9	0.0371 (10)	0.0357 (9)	0.0355 (10)	0.0080 (8)	0.0059 (8)	0.0157 (8)
N12	0.0478 (9)	0.0414 (8)	0.0337 (8)	0.0040 (7)	0.0104 (7)	0.0130 (7)
C12	0.0356 (9)	0.0359 (9)	0.0345 (10)	0.0071 (7)	0.0074 (8)	0.0157 (8)
N3	0.0419 (9)	0.0375 (8)	0.0465 (9)	0.0002 (7)	−0.0005 (7)	0.0184 (7)
C4	0.0376 (10)	0.0399 (10)	0.0386 (10)	−0.0003 (8)	0.0084 (8)	0.0162 (8)
N6	0.0510 (10)	0.0433 (9)	0.0371 (8)	0.0113 (7)	0.0055 (7)	0.0184 (7)
N9	0.0410 (9)	0.0636 (11)	0.0379 (9)	0.0014 (8)	0.0068 (7)	0.0257 (8)
C14	0.0378 (10)	0.0345 (9)	0.0340 (9)	0.0101 (8)	0.0042 (8)	0.0158 (8)
C3	0.0355 (10)	0.0417 (10)	0.0388 (10)	0.0050 (8)	0.0065 (8)	0.0206 (8)
C13	0.0381 (10)	0.0469 (11)	0.0361 (10)	0.0085 (8)	0.0051 (8)	0.0211 (9)
C6	0.0315 (9)	0.0438 (10)	0.0340 (9)	0.0060 (8)	0.0092 (7)	0.0162 (8)
C10	0.0392 (11)	0.0532 (12)	0.0458 (11)	0.0014 (9)	0.0072 (9)	0.0170 (10)
C11	0.0526 (12)	0.0416 (10)	0.0386 (10)	−0.0013 (9)	0.0072 (9)	0.0152 (9)
C1	0.0326 (10)	0.0471 (11)	0.0675 (14)	0.0022 (9)	−0.0023 (9)	0.0262 (10)
C2	0.0467 (12)	0.0517 (12)	0.0385 (11)	0.0049 (9)	0.0006 (9)	0.0155 (9)
C15	0.0535 (13)	0.0769 (15)	0.0432 (11)	0.0097 (11)	0.0124 (10)	0.0345 (11)
C7	0.0627 (14)	0.0434 (11)	0.0638 (14)	0.0043 (10)	0.0048 (11)	0.0257 (11)
C8	0.0726 (15)	0.0491 (12)	0.0582 (13)	0.0209 (11)	0.0138 (12)	0.0271 (11)
C16	0.0711 (15)	0.0603 (13)	0.0410 (11)	0.0120 (11)	0.0205 (11)	0.0215 (10)

### Geometric parameters (Å, º)

**Table:**

O2—C5	1.221 (2)	N6—H6	0.8600
O4—C14	1.221 (2)	N9—C13	1.327 (2)
O3—C13	1.221 (2)	N9—C15	1.447 (2)
O1—C4	1.217 (2)	N9—H9	0.8600
N11—C12	1.280 (2)	C3—C1	1.487 (2)
N11—N10	1.4251 (19)	C6—C2	1.491 (2)
N5—C6	1.274 (2)	C10—H10A	0.9600
N5—N4	1.418 (2)	C10—H10B	0.9600
N8—C9	1.276 (2)	C10—H10C	0.9600
N8—N7	1.423 (2)	C11—H11A	0.9600
N1—C6	1.400 (2)	C11—H11B	0.9600
N1—C4	1.413 (2)	C11—H11C	0.9600
N1—N2	1.423 (2)	C1—H1A	0.9600
N4—C3	1.388 (2)	C1—H1B	0.9600
N4—C5	1.414 (2)	C1—H1C	0.9600
N2—C3	1.276 (2)	C2—H2A	0.9600
N10—C9	1.386 (2)	C2—H2B	0.9600
N10—C14	1.404 (2)	C2—H2C	0.9600
N7—C12	1.390 (2)	C15—H15A	0.9600
N7—C13	1.406 (2)	C15—H15B	0.9600
C5—N6	1.325 (2)	C15—H15C	0.9600
C9—C10	1.491 (2)	C7—H7A	0.9600
N12—C14	1.328 (2)	C7—H7B	0.9600
N12—C16	1.451 (2)	C7—H7C	0.9600
N12—H12	0.8600	C8—H8A	0.9600
C12—C11	1.489 (2)	C8—H8B	0.9600
N3—C4	1.323 (2)	C8—H8C	0.9600
N3—C7	1.445 (2)	C16—H16A	0.9600
N3—H3	0.8600	C16—H16B	0.9600
N6—C8	1.437 (3)	C16—H16C	0.9600
			
C12—N11—N10	114.96 (14)	N5—C6—C2	117.15 (17)
C6—N5—N4	115.19 (15)	N1—C6—C2	122.75 (16)
C9—N8—N7	115.02 (15)	C9—C10—H10A	109.5
C6—N1—C4	124.52 (15)	C9—C10—H10B	109.5
C6—N1—N2	116.29 (14)	H10A—C10—H10B	109.5
C4—N1—N2	114.69 (14)	C9—C10—H10C	109.5
C3—N4—C5	124.12 (14)	H10A—C10—H10C	109.5
C3—N4—N5	116.73 (14)	H10B—C10—H10C	109.5
C5—N4—N5	115.63 (14)	C12—C11—H11A	109.5
C3—N2—N1	114.98 (15)	C12—C11—H11B	109.5
C9—N10—C14	125.66 (14)	H11A—C11—H11B	109.5
C9—N10—N11	117.72 (13)	C12—C11—H11C	109.5
C14—N10—N11	115.71 (14)	H11A—C11—H11C	109.5
C12—N7—C13	124.70 (15)	H11B—C11—H11C	109.5
C12—N7—N8	117.36 (14)	C3—C1—H1A	109.5
C13—N7—N8	114.98 (14)	C3—C1—H1B	109.5
O2—C5—N6	124.99 (17)	H1A—C1—H1B	109.5
O2—C5—N4	120.13 (16)	C3—C1—H1C	109.5
N6—C5—N4	114.86 (15)	H1A—C1—H1C	109.5
N8—C9—N10	120.67 (16)	H1B—C1—H1C	109.5
N8—C9—C10	116.79 (17)	C6—C2—H2A	109.5
N10—C9—C10	122.46 (16)	C6—C2—H2B	109.5
C14—N12—C16	120.73 (16)	H2A—C2—H2B	109.5
C14—N12—H12	119.6	C6—C2—H2C	109.5
C16—N12—H12	119.6	H2A—C2—H2C	109.5
N11—C12—N7	120.50 (15)	H2B—C2—H2C	109.5
N11—C12—C11	117.34 (16)	N9—C15—H15A	109.5
N7—C12—C11	122.13 (15)	N9—C15—H15B	109.5
C4—N3—C7	121.39 (16)	H15A—C15—H15B	109.5
C4—N3—H3	119.3	N9—C15—H15C	109.5
C7—N3—H3	119.3	H15A—C15—H15C	109.5
O1—C4—N3	124.76 (18)	H15B—C15—H15C	109.5
O1—C4—N1	120.22 (17)	N3—C7—H7A	109.5
N3—C4—N1	114.97 (15)	N3—C7—H7B	109.5
C5—N6—C8	120.58 (16)	H7A—C7—H7B	109.5
C5—N6—H6	119.7	N3—C7—H7C	109.5
C8—N6—H6	119.7	H7A—C7—H7C	109.5
C13—N9—C15	120.93 (16)	H7B—C7—H7C	109.5
C13—N9—H9	119.5	N6—C8—H8A	109.5
C15—N9—H9	119.5	N6—C8—H8B	109.5
O4—C14—N12	124.41 (16)	H8A—C8—H8B	109.5
O4—C14—N10	120.80 (16)	N6—C8—H8C	109.5
N12—C14—N10	114.76 (15)	H8A—C8—H8C	109.5
N2—C3—N4	120.34 (16)	H8B—C8—H8C	109.5
N2—C3—C1	117.75 (17)	N12—C16—H16A	109.5
N4—C3—C1	121.85 (16)	N12—C16—H16B	109.5
O3—C13—N9	124.37 (17)	H16A—C16—H16B	109.5
O3—C13—N7	120.74 (17)	N12—C16—H16C	109.5
N9—C13—N7	114.83 (16)	H16A—C16—H16C	109.5
N5—C6—N1	120.09 (15)	H16B—C16—H16C	109.5
			
C6—N5—N4—C3	33.9 (2)	C6—N1—C4—N3	162.35 (16)
C6—N5—N4—C5	−166.36 (15)	N2—N1—C4—N3	7.1 (2)
C6—N1—N2—C3	33.9 (2)	O2—C5—N6—C8	−5.5 (3)
C4—N1—N2—C3	−168.75 (16)	N4—C5—N6—C8	172.64 (16)
C12—N11—N10—C9	31.0 (2)	C16—N12—C14—O4	1.3 (3)
C12—N11—N10—C14	−159.33 (16)	C16—N12—C14—N10	179.32 (17)
C9—N8—N7—C12	32.6 (2)	C9—N10—C14—O4	−8.9 (3)
C9—N8—N7—C13	−165.91 (16)	N11—N10—C14—O4	−177.71 (15)
C3—N4—C5—O2	−25.0 (3)	C9—N10—C14—N12	172.96 (16)
N5—N4—C5—O2	176.99 (15)	N11—N10—C14—N12	4.2 (2)
C3—N4—C5—N6	156.80 (16)	N1—N2—C3—N4	−4.4 (2)
N5—N4—C5—N6	−1.2 (2)	N1—N2—C3—C1	178.52 (17)
N7—N8—C9—N10	−6.1 (2)	C5—N4—C3—N2	172.45 (16)
N7—N8—C9—C10	177.08 (15)	N5—N4—C3—N2	−29.7 (2)
C14—N10—C9—N8	165.74 (17)	C5—N4—C3—C1	−10.6 (3)
N11—N10—C9—N8	−25.7 (2)	N5—N4—C3—C1	147.23 (18)
C14—N10—C9—C10	−17.6 (3)	C15—N9—C13—O3	1.0 (3)
N11—N10—C9—C10	150.96 (17)	C15—N9—C13—N7	178.38 (17)
N10—N11—C12—N7	−4.4 (2)	C12—N7—C13—O3	−19.3 (3)
N10—N11—C12—C11	177.61 (16)	N8—N7—C13—O3	−179.19 (17)
C13—N7—C12—N11	173.17 (17)	C12—N7—C13—N9	163.24 (17)
N8—N7—C12—N11	−27.3 (2)	N8—N7—C13—N9	3.3 (2)
C13—N7—C12—C11	−8.9 (3)	N4—N5—C6—N1	−4.2 (2)
N8—N7—C12—C11	150.58 (17)	N4—N5—C6—C2	176.52 (15)
C7—N3—C4—O1	4.1 (3)	C4—N1—C6—N5	175.41 (16)
C7—N3—C4—N1	−178.51 (17)	N2—N1—C6—N5	−29.7 (2)
C6—N1—C4—O1	−20.2 (3)	C4—N1—C6—C2	−5.3 (3)
N2—N1—C4—O1	−175.36 (16)	N2—N1—C6—C2	149.51 (17)

### Hydrogen-bond geometry (Å, º)

**Table:**

*D*—H···*A*	*D*—H	H···*A*	*D*···*A*	*D*—H···*A*
N3—H3···N2	0.86	2.16	2.572 (2)	108
N6—H6···N5	0.86	2.19	2.586 (2)	108
N9—H9···N8	0.86	2.15	2.567 (2)	109
N12—H12···N11	0.86	2.17	2.581 (2)	109
N3—H3···O4	0.86	2.20	2.925 (2)	142
N6—H6···O3^i^	0.86	2.16	2.918 (2)	146
N9—H9···O1^ii^	0.86	2.14	2.877 (3)	143
N12—H12···O2^iii^	0.86	2.24	2.967 (3)	142

Symmetry codes: (i) *x*−1, *y*, *z*−1; (ii) −*x*+1, −*y*+1, −*z*+1; (iii) −*x*+2, −*y*+2, −*z*+1.
